#  Exposure to Secondhand Cannabis Smoke Among Children

**DOI:** 10.1001/jamanetworkopen.2024.55963

**Published:** 2025-01-23

**Authors:** Osika Tripathi, Humberto Parada, Connie Sosnoff, Georg E. Matt, Penelope J. E. Quintana, Yuyan Shi, Sandy Liles, Lanqing Wang, Kevin T. Caron, James Oneill, Ben Nguyen, Benjamin C. Blount, John Bellettiere

**Affiliations:** 1 San Diego State University, School of Public Health, San Diego, California; 2 Herbert Wertheim School of Public Health and Human Longevity Science, University of California, San Diego; 3 San Diego State University, Department of Psychology, San Diego, California; 4 Tobacco and Volatiles Branch, Division of Laboratory Sciences, National Center for Environmental Health, Centers for Disease Control and Prevention, Atlanta, Georgia; 5 San Diego State University, Department of Mechanical Engineering, San Diego, California

## Abstract

**Importance:**

The degree that in-home cannabis smoking can be detected in the urine of resident children is unclear.

**Objective:**

Test association of in-home cannabis smoking with urinary cannabinoids in children living at home.

**Design, Setting, and Participants:**

This cross-sectional study used baseline data from Project Fresh Air, a 2012-2016 randomized clinical trial to reduce fine particulate matter levels. Eligible participants were recruited from households in San Diego County, California, with children under age 14 years and an adult tobacco smoker in residence. Children’s urine samples were analyzed in 2022.

**Exposures:**

In-home cannabis smoking, measured by: parent or guardian report of in-home cannabis smoking; number of daily nonspecific smoking events computed via an air particle count algorithm; and number of daily cannabis smoking events ascertained by residualization, adjusting for air nicotine, tobacco smoking, and other air particle generating or ventilating activities.

**Main Outcomes and Measures:**

Levels of the cannabis biomarker Δ^9^-tetrahydrocannabinol (THC) and its major metabolites, 11-hydroxy-Δ^9^-tetrahydrocannabinol and 11-nor-9-carboxy-Δ^9^-tetrahydrocannabinol. Biomarker molar equivalents were summed to represent total THC equivalents (TTE) in urine. Logistic regression assessed whether in-home smoking was associated with cannabis biomarker detection. For children with detectable urinary cannabinoids, linear regression assessed in-home smoking association with quantity of urinary TTE.

**Results:**

A total of 275 children were included in analysis (mean [SD] age, 3.6 [3.6] years; 144 male [52.4%]; 38 Black [13.8%], 132 Hispanic [48.0%], and 52 White [18.9%]). Twenty-nine households (10.6%) reported in-home cannabis smoking in the past 7 days; 75 children [27.3%] had detectable urinary cannabinoids. Odds of detectable TTE in children’s urine were significantly higher in households with reported in-home cannabis smoking than households without (odds ratio [OR], 5.0; 95% CI, 2.4-10.4) and with each additional ascertained daily cannabis smoking event (OR, 2.5; 95% CI, 1.6-3.9). Although the point estimate for TTE levels was higher among children with detectable urinary cannabinoids and exposure to more daily cannabis smoking events (increase per event, 35.68%; 95% CI, −7.12% to 98.21%), the difference was not statistically significant.

**Conclusions and Relevance:**

In this cross-sectional study, in-home cannabis smoking was associated with significantly increased odds of child exposure to cannabis smoke, as assessed by urinary cannabinoid biomarkers. As young children spend most of their time at home, reducing in-home cannabis smoking could substantially reduce their exposure to the toxic and carcinogenic chemicals found in cannabis smoke.

## Key Points

**Question:**

How is cannabis smoking in the home associated with resident children’s exposure to cannabis smoke, as measured by urinary biomarkers?

**Findings:**

In this cross-sectional study of 275 children, the odds of detectable THC equivalents in children’s urine were 5 times higher in households with reported in-home cannabis smoking compared with households without.

**Meaning:**

These results suggest that reducing in-home cannabis smoking could substantially reduce children’s exposure to cannabis smoke, which contains toxic chemicals including known carcinogens.

## Teaser

This cross-sectional study of households in California examines the association of in-home cannabis smoking with urinary cannabinoids in children living at home.

## Introduction

The most common method of cannabis use, smoking,^1^ is known to generate emissions^2,3,4^ that are harmful to those exposed. Cannabis is often smoked indoors,^5^ and nonsmokers such as children are at risk for exposure, even more so now that cannabis use is on the rise among parents with children living at home.^6,7,8^ While the long-term health consequences of exposure to cannabis smoke are not yet well known,^9^ cannabis smoke contains carcinogens, respiratory irritants, and other harmful chemicals.^2,3,4^ Cannabis smoking also emits large amounts of PM_2.5_ (fine particulate matter with aerodynamic diameters 2.5 μm or below), which negatively impacts cardiovascular and pulmonary health^10^ and correlates with respiratory burden of carbon monoxide and insoluble particulates (tar).^4^ Secondhand cannabis smoke (SHCS) is smoke either directly from burned cannabis or from smoker exhalations. SHCS exposure has only recently been objectively quantified through detection of small amounts of cannabinoids in the urine.^11,12,13,14,15^

SHCS exposure is often involuntary and those exposed may be especially vulnerable to adverse health outcomes. One small study found child exposure to indoor cannabis smoke to increase the likelihood of reporting adverse health outcomes (emergency department visits, ear infections, bronchitis or bronchiolitis, asthma, or skin conditions) when compared with children with no such exposure), although these results were not statistically significant.^16^ Another study found children having caregivers who smoked cannabis had higher rates of viral respiratory infections.^17^ Some studies reported a positive association of adverse or problematic cognitive, emotional, or mental health outcomes with pre- and postnatal cannabis smoke exposure.^18,19,20^ Given children’s vulnerability to environmental exposures,^21,22,23^ studying how their cannabis smoke exposure can be decreased is important.

The principal psychoactive constituent of cannabis is (-)-trans-Δ^9^-tetrahydrocannabinol (THC). In the human body, THC is metabolized to 11-hydroxy-Δ^9^-tetrahydrocannabinol (OH-THC), and OH-THC is further metabolized to 11-nor-9-carboxy-Δ^9^-tetrahydrocannabinol (COOH-THC).^24^ All 3 cannabinoids, THC, OH-THC, and COOH-THC, can be measured in urine and can be used as biomarkers for cannabis exposure.^14^

While a few studies have described COOH-THC in children’s urine and parental report of cannabis smoking around the children,^11,12^ they did not quantify the relationship between in-home cannabis smoking and urinary cannabis biomarkers. These studies depended exclusively on the measurement of urinary COOH-THC, the primary metabolite of THC in urine. Additional biomarkers of exposure, such as THC and OH-THC, may help produce better estimates of total exposure. This study assessed the association between in-home cannabis smoking and SHCS exposure in resident children as assessed by urinary cannabinoid levels (THC, OH-THC, and COOH-THC).

## Methods

### Study Design

Project Fresh Air (PFA) was a 2-group randomized clinical intervention aimed at reducing indoor fine particulate matter (PM_0.5–2.5_) levels through real-time feedback and coaching in San Diego County, California.^25,26^ This secondary cross-sectional study, Eliminating In-Home Smoking (EIS), funded in 2020, used deidentified data collected during an approximately 7-day period before PFA intervention commencement. The University of California, San Diego institutional review board certified that our study presented no human participants protection concerns. Written informed consent was obtained from each participant in Project Fresh Air using a consent form approved by the San Diego State University institutional research board. This study followed the Strengthening the Reporting of Observational Studies in Epidemiology (STROBE) reporting guideline.

### Study Participants

PFA recruited participants May 2012 through December 2015 through various sources, including community events and organizations such as the Women, Infants, and Children nutrition programs. Study inclusion criteria were age 18 years or older, having at least 1 child under age 14 years living in the home, having a resident adult tobacco smoker, and having no plans of moving for 3 months. We sequentially enrolled 298 households, 1 parent or guardian and the youngest child from each household, with each participating in the trial for approximately 3 to 4 months during October 2012 to February 2016. Details of participant recruitment and enrollment are reported elsewhere.^25^ Of 298 children enrolled, at baseline 10 had no stored urine samples, 2 lacked all cannabinoid data due to assay issues, 10 were missing air particle data, and 1 participant’s data were excluded due to extreme cannabis biomarker values (THC, 8.0 ng/mL; OH-THC, 168.0 ng/mL; COOH-THC, 331.0 ng/mL) consistent with primary exposure to cannabis. The final analytic sample was 275 households (Figure).

**Figure.**
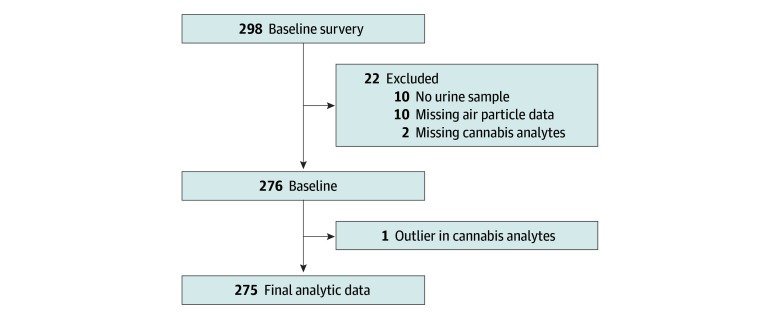
Flowchart for the Analytic Dataset

### Study Procedures

After study enrollment, project staff installed an air particle monitor (Dylos) in the room where tobacco smoking most commonly occurrred.^27^ The monitor continuously counted fine air particles (0.5 to 2.5 μm in diameter). Passive air nicotine dosimeters were also placed within 2 feet of the air particle monitor. Approximately 7 days after installing the particle monitor, project staff administered a face-to-face computer-assisted interview with the enrolled parent or guardian to assess participant and household characteristics and air particle generating activities that occurred in the past 7 days.

The parent or guardian was provided a urine collection kit, including verbal, written, and pictorial instructions on how to collect urine samples from the enrolled child. Urine samples were collected within 24 hours of the visit and were transported by project staff to the biosafety laboratory. Contaminated samples (eg, containing feces or baby powder) were discarded, and another sample was collected. Valid urine samples were stored in lab freezers at or below −20 °C. In July and August 2020, at the very beginning of the EIS study, archived urine aliquots were sent frozen to the Centers for Disease Control and Prevention (CDC), National Center for Environmental Health, Tobacco and Volatiles Branch for analysis of cannabis exposure biomarkers.

### Measures of In-Home Cannabis Smoking

In-home cannabis smoking was measured using data from air particle monitoring and reported in-home cannabis smoking in the past 7 days, concurrent with the air particle monitor data collection period. The air particle monitor produced a sensitive measure of smoking and other air particle generating activities in the home but did not identify the source of air particles. Reported in-home cannabis smoking had high specificity but may have false negatives that can underestimate true exposure. To assess in-home cannabis smoking, parents and guardians were asked, “How often in the past 7 days did anyone smoke medicinal or recreational marijuana in your home?” The responses were then binary coded as “no” for households with zero times and “yes” when reporting 1 time or more. To assess number of daily nonspecific smoking events, we used particle counts from air monitors, using a previously validated algorithm from Hovell et al (information on the algorithm available in the study supplement).^26^ Monitors counted particles irrespective of their source (eg, smoking cigarettes, smoking cannabis, burning toast, cooking with oil, burning incense). Daily smoking events were identified if the maximum particle count was 15 000 counts/0.01 ft^3^ or above within a 5-minute period.

### Measures of Cannabis Exposure

At CDC’s Tobacco and Volatiles Branch, urine samples were first hydrolyzed with β-glucuronidase to free the bound analytes, then a panel of 3 cannabis biomarkers (THC, OH-THC, and COOH-THC) were measured by isotope-dilution ultrahigh performance liquid chromatography-electrospray ionization tandem mass spectrometry based on modification of the validated method of Wei et al.^14^ The limit of detection (LOD) for each biomarker was 0.005 ng/mL. Values below LOD were estimated as half LOD (0.0025 ng/mL).

The molar equivalents of THC, OH-THC, and COOH-THC were summed to create a cumulative measure of exposure, total THC equivalents (TTE, measured in nmol/L). For those detectable for at least 1 biomarker, TTE was calculated as the sum of detectable values plus 0.0025 ng/mL for undetectable metabolites. We created a binary TTE variable, coded “not detected” if undetectable for all 3 biomarkers, and coded “detected” if detectable for 1 or more biomarker;. Each biomarker was also coded according to whether that urinary metabolite had a detectable value.

### Covariates

Covariates included household demographic variables (sex and age of child, parent or guardian’s education, family income, race and ethnicity, and type of home), in-home tobacco smoking variables, report of particle generating (eg, use of incense, gas or propane appliances) and ventilation (eg, use of air purifier, exhaust fan) activities, and nicotine dosimeter assay (eMethods in Supplement 1). To capture the diversity of our lower income sample, investigators categorized race and ethnicity as Black, Hispanic, White, and other (including Asian or Pacific Islander, American Indian or Alaska Native, multiple races, and unspecified).

### Statistical Analysis

Descriptive statistics of the 275 households included in the EIS study were computed. Missing data were highest for reported in-home cannabis smoking (77 of 275 [28.0%]), followed by reported in-home cigarette, cigar, pipe, hookah, e-cigarette smoking (34 of 275 [12.4%]), family income (28 of 275 [10.2%]), and air nicotine (12 of 275 [4.4%]). To minimize bias due to missing data, we used Multiple Imputation by Chained Equations (MICE) implemented through the MICE package in R (R Project for Statistical Computing),^28,29^ generating 30 imputations, each with 50 iterations. We used the default method of imputation, which differs by variable class, and the method of estimation was bayesian. This assumes that data are missing at random and uses an iterative algorithm to generate plausible values for missing entries. The imputation model included all covariates including variables described in eMethods in Supplement 1. All analyses beyond descriptive statistics used multiple imputed data.

We used multiple variables to estimate in-home cannabis smoking, recognizing that no single variable offers an optimal estimate. Consequently, we applied the residualization approach,^30,31^ combining the specificity of self-reported in-home cannabis smoking with the sensitivity of objective air particle measurements.

#### In-Home Cannabis Smoking Ascertainment Via a Residualization^31^ Approach

To partition out the amount of objective air particle data related to reported in-home cannabis smoking, linear regression was conducted with number of daily nonspecific smoking events as the dependent variable, and all reported in-home tobacco smoking, in-home air particle generating and ventilation activities, and air nicotine as the independent variables. The residuals from this model (model A) provided information on variance in the air particle data not due to the variables specified in model A (eTable 1 in Supplement 1). A second model (model B) was estimated with the addition of reported in-home cannabis smoking to the model. The residuals from model B provided information on variance in the air particle data not due to any of the specified air particle generating behaviors. The difference in the residuals from the 2 models (residuals of model A minus residuals of model B) equals the variance in the number of daily smoking events uniquely attributed to reported in-home cannabis smoking, termed the *ascertained* number of daily cannabis smoking events. A detailed guide on the computation of ascertained number of daily cannabis smoking events is available in eTable 1 in Supplement 1.

#### Primary Analyses—Association of In-Home Cannabis Smoking With Urinary Cannabinoids in Children

To model the association of in-home cannabis smoking with urinary cannabinoids, 2 multivariable models were examined: a logistic regression model estimated the odds of cannabis biomarkers being detected in the children’s urine (model 1); among children with detectable cannabis biomarkers, a linear regression model quantified the effect size of frequency of in-home cannabis smoking with TTE (model 2). In these linear models, outcome variables were natural log–transformed to address the non-normal distributions. The final model (model 3) is adjusted for all household demographic variables.

We evaluated the linearity of number of daily nonspecific smoking events and ascertained number of daily cannabis smoking events using restricted cubic splines (RCS). Number of daily nonspecific smoking events exhibited statistically significant nonlinearity (nonlinear *P* < .05), while ascertained number of daily cannabis smoking events did not (nonlinear *P* > .14). We modeled number of daily nonspecific smoking events with 4 knots using the fully adjusted logistic model (model 3) fit within the multiple imputation framework and Rubin rules to pool adjusted predictions and variances across imputations. We plotted the results with the referent category set to zero, outputting predictions for the zero through 99th percentile to facilitate interpretation (eFigure 1 in Supplement 1). We also included logistic regression estimates when reporting TTE because they are informative for most households, given the association appears linear up to approximately 10 events, representing 225 of 275 households (81.2%) in this study.

#### Sensitivity Analyses

We conducted stratified logistic regression by children’s age (under age 6 years vs 6 years and older) to identify important differences by activity patterns and locations.^21,22,23^ As 208 of 275 children (75.6%) were under the age of 6 years, the results among children ages 6 years and older may not be reliable due to the small sample size.

## Results

A total of 275 children were analyzed (mean [SD] age, 3.6 [3.6] years; 144 male [52.4%]; 38 Black [13.8%], 132 Hispanic [48.0%], 52 White [18.9%]) (Table 1). One-fifth of the households had an annual family income below $10 000 (53 households [19.2%]), and the mean (SD) level of education of parents or guardians was 13.2 (3.3) years.

**Table 1.** Descriptive Statistics for Questionnaire, Air Particle, and Biomarker Variables

Characteristic	Participants, No. (%) (N = 275)
Cannabis smoked inside home in past 7 d^a^	
No	169 (61.5)
Yes	29 (10.6)
Missing	77 (28.0)
Number of daily nonspecific smoking events^b^	
Mean (SD)	2.9 (5.0)
Median (range)	0.9 (0-33.6)
Total THC equivalents, nmol/L^c^^,^^d^^,^^e^	
Not detected	200 (72.7)
Detected	75 (27.3)
Mean (SD) [half LOD]^f^	0.13 (0.42)
Median (range) [half LOD]^f^	0.02 (0.02-4.95)
THC, ng/mL^c^	
Not detected	251 (91.3)
Detected	24 (8.7)
OH-THC, ng/mL^c^	
Not detected	218 (79.3)
Detected	54 (19.6)
Missing	3 (1.1)
COOOH-THC, ng/mL^c^	
Not detected	209 (76.0)
Detected	62 (22.6)
Missing	4 (1.5)
Age of child, y	
Mean (SD)	3.6 (3.6)
Median (range)	3.0 (0-14.0)
Sex of child^a^	
Female	131 (47.6)
Male	144 (52.4)
Race or ethnicity of child^a^	
Black	38 (13.8)
Hispanic	132 (48.0)
White	52 (18.9)
Other	53 (19.3)
Family income (annual)^a^	
<$10 000	53 (19.2)
$10 000-$19 999	44 (16.0)
$20 000-$29 999	46 (16.7)
$30 000-$39 999	32 (11.6)
$40 000-$49 999	26 (9.5)
≥$50 000	45 (16.3)
Missing	29 (10.6)
Education level of parent or guardian, y^a^	
Mean (SD)	13.2 (3.3)
Median (range)	13.0 (0.7-22.0)
Home type^a^	
Apartment or condominium	114 (41.5)
Detached house	119 (43.3)
Other	42 (15.3)

Abbreviations: COOH-THC, 11-nor-9-carboxy-Δ^9^-tetrahydrocannabinol; LOD, limit of detection; OH-THC, 11-hydroxy-Δ^9^-tetrahydrocannabinol.

^a^ Reported by parent or guardian; other race and ethnicity category comprises Asian or Pacific Islander, American Indian or Alaska Native, multiple races, and unspecified.

^b^ Determined by a validated air particle count algorithm, using data from a wide variety of air particle sources (eg, smoking cigarettes, smoking cannabis, burning toast, cooking with oil, burning incense).

^c^ Cannabis biomarker data.

^d^ Each biomarker (ng/mL) was divided by its molecular weight (ng/nmol) to yield nmol/mL and summed: *TTE = (THC [ng/mL] / 314.5 [ng/nmol]) + (OH-THC [ng/ml] / 330.5 [ng/nmol]) + (COOH-THC [ng/mL]) / 344.4 [ng/nmol])*.

^e^ For the categorization of TTE, those with undetectable LOD for all 3 biomarkers were considered ‘not detected’.

^f^ Half LOD = 0.0025 ng/mL.

Participating households had a mean (SD) 2.9 (5.0) daily smoking events. Parents or guardians of 29 households (10.6%) reported that cannabis was smoked inside their home in the past 7 days; 75 children (27.3%) had detectable cannabis exposure biomarkers in their urine. The mean (SD) TTE was 0.13 (0.42) nmol/L. Detailed descriptive statistics can be found in Table 1 and eTable 2 in Supplement 1. Among households reporting in-home cannabis smoking, 20 of 29 children (69.0%) had detectable TTE levels compared with 40 of 169 (23.7%) in households without report of in-home cannabis smoking.

### Smoking Events

The household demographics-adjusted odds of detecting cannabis biomarkers in children’s urine among households that reported in-home cannabis smoking in the past 7 days were 5.02 times (95% CI, 2.42-10.40) the odds of households without reported in-home cannabis smoking (Table 2).

**Table 2.** Estimates From Logistic Regression of Total Detectable THC Equivalents (TTE) in Children and Linear Regression of TTE Among Children With Detectable Urinary Cannabinoids^a^

Model^b^	Logistic, OR (95% CI) (N = 275)	Log-linear, % (95% CI) (n = 75)
**In-home cannabis smoking in past 7 d** ^c^		
1	5.36 (2.71 to 10.61)	80.82 (−0.42 to 228.34)
2	4.85 (2.34 to 10.03)	76.44 (−4.05 to 224.46)
3	5.02 (2.42 to 10.40)	86.11 (−1.42 to 251.35)
**No. of daily nonspecific smoking events** ^d^		
1	1.13 (1.07 to 1.19)	6.55 (2.12 to 11.17)
2	1.10 (1.04 to 1.17)	4.89 (−0.11 to 10.13)
3	1.10 (1.04 to 1.17)	4.72 (−0.44 to 10.16)
**Ascertained number of daily cannabis smoking events** ^e^		
1	2.32 (1.52 to 3.55)	20.43 (−15.80 to 72.23)
2	2.49 (1.59 to 3.91)	32.06 (−8.61 to 90.82)
3	2.50 (1.59 to 3.92)	35.68 (−7.12 to 98.21)

Abbreviations: LOD, limit of detection; OR, odds ratio; THC, tetrahydrocannabinol.

^a^ For both procedures, detectability of TTE was determined based on cannabis biomarkers below the LOD treated as half of LOD (0.0025 ng/mL).

^b^ Model 1 was the unadjusted model; model 2, model 1 plus the sex of the child (male or female), age of the child (years), parent or guardian’s education (years), family income (increments of $10 000), race and ethnicity (Black, White, Hispanic, other [Asian or Pacific Islander, American Indian or Alaska Native, multiple, and unspecified]); model 3, model 2 plus type of home (apartment or condominium, detached house, or other).

^c^ Reported by parent or guardian.

^d^ Determined by a validated air particle count algorithm using air particle data nonspecific to the source of air particles (eg, smoking cigarettes, smoking cannabis, burning toast, cooking with oil, burning incense).

^e^ Ascertained by residualization, adjusting for air nicotine, tobacco smoking, and other air reported particle generating or ventilating activities.

The household demographics-adjusted odds of detecting cannabis biomarkers in children’s urine increased by a factor of 1.10 (95% CI, 1.04-1.17) for each additional daily in-home nonspecific smoking event up to 10 events (Table 2). Although the point estimate for TTE levels was higher among children with detectable urinary cannabinoids and exposure to more daily smoking events (increase per event, 4.72%; 95% CI, −0.44% to 10.16%), the difference was not statistically significant.

For every ascertained daily cannabis smoking event, the household demographics-adjusted odds of cannabis biomarkers being detected in the children’s urine increased statistically significantly by a factor of 2.50 (95% CI, 1.59-3.92) (Table 2). Although the point estimate for TTE levels was higher among children with detectable urinary cannabinoids and exposure to more daily cannabis smoking events (increase per ascertained daily cannabis smoking event, 35.68%; 95% CI, −7.12% to 98.21%), the difference was not statistically significant.

In an additional analysis, we compared households with and without missing data for in-home cannabis smoking in the last 7 days due to the substantial proportion of missing data (77 households [28.0%]) for this key variable (eTables 5 and 6 in Supplement 1). Households with missing data had nonsignificant lower levels of TTE detection (15 of 77 [19.5%]) than households without missing data (60 of 198 [30.3%]) (*P* = .10) (eTable 5 in Supplement 1).

### Sensitivity Analyses Results

The logistic regression estimates between the 2 age groups of children varied greatly. However, among children ages 6 years and younger, the estimate for ascertained number of daily cannabis smoking events (OR, 2.17; 95% CI, 1.29-3.65) was similar to that of the overall sample (eTable 4 in Supplement 1).

## Discussion

Through the application of 3 distinct measures of cannabis smoking—subjective (self-report), objective (air particle monitor data), and statistically ascertained (integrating self-report and air particle monitor data)—our findings demonstrate a consistent association between in-home cannabis smoking and biological markers (evidenced in urine samples) of SHCS exposure in children residing within these households. Research on cannabis lags more than 50 years behind that of tobacco due to restrictive policies and regulations around cannabis research.^32^ There are limited data assessing long-term cannabis smoking health effects, and even fewer assessing SHCS exposure effects.^33^ A 2017 study^11^ of young children (aged 1 month to 2 years) who were hospitalized for bronchiolitis in Colorado from 2013 to 2015 found 16% had detectable COOH-THC and 16% reported cannabis use by a caregiver in the home. In a 2021 study^12^ of children aged zero to 3 years who had well-child appointments in the hospital or were in an inpatient unit (in 2017 and 2018), 22% had detectable COOH-THC and 15% of caregivers reported cannabis use by a caregiver in the home.^12^ In comparison, 23% of the children in our study had detectable levels of COOH-THC and 11% of parents or guardians reported cannabis was smoked inside the home recently.

As evidence regarding the health effects of cannabis grows, adopting strategies from the tobacco control playbook—eg, comprehensive smoke-free laws and policies for homes, vehicles, workplaces, and public spaces prohibiting indoor use at all times—could safeguard children’s health. Among US adults who reported smoking cannabis in the past year, 70% lacked set rules against indoor cannabis smoking in their homes; notably, 13% of these individuals resided with children under age 6 years.^34^ The adoption and strict enforcement of household rules forbidding indoor smoking have been shown to deter tobacco smoking within homes with children.^35,36,37^ Similar measures could be equally effective in preventing cannabis smoking indoors.

### Strengths and Limitations

Our study offers unique contributions to the cannabis control field by examining the association between in-home cannabis smoking and children’s SHCS exposure. Not only did we use the state-of-the-art ascertainment of cannabis exposure (urinary biomarkers), we calculated the molar sum (TTE) of 3 urinary cannabis biomarkers (THC, OH-THC, and COOH-THC) to provide a more sensitive estimate of exposure than would measurement of a single biomarker. But as a high percentage of cannabinoids are excreted in feces, our measure may still underestimate exposure.^24^ Furthermore, we used 3 measures of in-home cannabis smoking including a newly developed metric that integrated objective air particle data and self-reported in-home cannabis smoking. This metric enabled us to partition out the portion of air particle data due to reported in-home cannabis smoking. We used 2 regression modeling approaches to determine both the likelihood of TTE detection and the quantity of TTE in those with detectable TTE. However, our analysis of the quantity of TTE was constrained to children with evidence of exposure, which limited our sample size and likely led to findings that were directional but not statistically significant.

Another methodological strength is the temporal alignment of our exposure and outcome measures. Urine samples, collected on the final day of the pretest week, aligned with the COOH-THC half-life averaging 6 to 7 days.^24,38^ Moreover, parental reports of in-home cannabis smoking, air particle measures, and air nicotine measures also covered that 7-day period.

Our study had several limitations. In-home cannabis smoking could be underreported due to social desirability bias—particularly as non–medical cannabis use was illegal in California when data were collected (2012 to 2016)—potentially underestimating associations between in-home cannabis smoking and child urinary cannabinoids. Nevertheless, our estimates were significant. PFA focused on tobacco, not cannabis, resulting in unmeasured factors that might impact children’s cannabis exposure. In-home cannabis smoking can vary in location and frequency (weekly to hourly). Cannabis smoking method, and level of PM_2.5_ emissions, differs from joints to blunts to bongs.^39^

Tobacco smoking is linked to cannabis use^8,40^; requiring that households include a tobacco-smoking resident may have increased proportions of in-home cannabis smoking in the study cohort. Earlier tendencies to smoke indoors to avoid detection of illegal cannabis use may have declined since 2016 due to more liberal current regulations, along with other changes in attitude and behaviors around cannabis. Further studies focused specifically on cannabis with more timely data are warranted.

The study was noncausal; ORs should not be interpreted as estimates of relative risk. PFA’s convenience sampling predominantly recruited participants from lower socioeconomic backgrounds and Hispanic ethnicity. This sample composition may limit generalizability of prevalence estimates from the study, although unlikely to impact the observed in-home smoking and child exposure association. Population-representative prevalence estimates of cannabis exposure would more authoritatively inform public health officials and researchers on this issue.

The first 33 enrolled homes were not administered questions about in-home cannabis smoking, but multiple imputation addressed missingness, while multiple measures of in-home smoking reduced bias in estimates. Additionally, we explored the differences between households with and without missing information on in-home cannabis smoking in the last 7 days, as well as running a complete data analysis among those with a response recorded for in-home cannabis smoking due to the substantial proportion of missing data (28.0%) for this key variable (eTables 5 and 6 in Supplement 1). Households with missing data have statistically insignificant lower levels of TTE detection. When compared with the logistic regression results on the complete data, our multiply imputed estimates were smaller, suggesting that our estimates were not overinflated due to any bias introduced by the missing data.

While detection of cannabinoids in our laboratory assays was likely primarily due to SHCS exposure of the participating child, detection could also have resulted from thirdhand smoke exposure or smoking mothers breastfeeding their child, factors not assessed in PFA. Exposure occurring outside the home may contribute to urinary cannabinoid levels, but the most common location of cannabis smoking seems to be at own or someone else’s home.^34^ Future cannabis studies should evaluate the effects of different locations and modes of use on cannabis exposure.

Lastly, the number of daily nonspecific smoking events had a nonlinear association with TTE after around 10 events. While future studies with larger sample sizes are needed to verify and further clarify any nonlinear relationships, we included a plot to show these results in our study. There is a slight dip in ORs below the threshold of significance for households below 1 daily nonspecific smoking event (eFigure 1 in Supplement 1), which we hypothesize resulted from the metric’s nonspecificity; ie, many homes have spurious events from other smoke-generation activities (like burning toast or candles^41^) that are not related to TTE. We also caution interpretation of these results for high-event homes (more than 10 nonspecific smoking events per day) given the sparse data in that range, which may have contributed to unstable estimates (evidenced by the wide CIs).

## Conclusions

Our study suggests in-home cannabis smoking and children’s SHCS exposure are related. Approaches to decrease in-home cannabis smoking, especially in households with children, should be tested. Investigation of how evolving cannabis regulatory environments impact in-home smoking patterns and potential increased child SHCS exposure is essential. Lastly, more information is needed on the long-term health risks of SHCS exposure.

## Notes

Supplement 1. **eMethods.** Questionnaire, Nicotine Dosimeter Data, and Sensitivity Analyses **eTable 1.** Models for Addressing Variance in Air Particle Data Due to Various Particle Generating Activities **eTable 2.** Descriptive Statistics for Reported Air Particle Generating and Ventilating Events and Air Nicotine **eTable 3.** Linear Regression for Total THC Equivalents Among Those With Detectable Urinary Cannabinoids **eTable 4.** Age-Stratified Logistic Regression of Total Detectable THC Equivalents in Urine of Children **eTable 5.** Descriptive Statistics for Demographic Variables Stratified by Missingness on Reported In-Home Cannabis Smoking **eTable 6.** Logistic Regression of Total Detectable THC Equivalents in Children, Among Those With Data on Reported In-Home Cannabis Smoking **eFigure 1.** Restricted Cubic Spline Regression between Number of Daily Nonspecific Smoking Events and Total THC Equivalents **eFigure 2.** Distribution of Log-TTE Among Households Reporting In-Home Cannabis Smoking Among Children With Detectable Levels of TTE **eFigure 3.** Distribution of Log-TTE by Number of Daily Nonspecific Smoking Events, Among Children With Detectable Levels of TTE **eFigure 4.** Distribution of Log-TTE by Ascertained Number of Daily Cannabis Smoking Events, Among Children With Detectable Levels of TTE

Supplement 2.
Data Sharing Statement

## References

[1] Shi Y. Heterogeneities in administration methods among cannabis users by use purpose and state legalization status: findings from a nationally representative survey in the United States, 2020. Addiction. 2021;116(7):1782-1793. doi:10.1111/add.15342 33217090 PMC8134617

[2] Moir D, Rickert WS, Levasseur G, . A comparison of mainstream and sidestream marijuana and tobacco cigarette smoke produced under two machine smoking conditions. Chem Res Toxicol. 2008;21(2):494-502. doi:10.1021/tx700275p 18062674

[3] Novotný M, Merli F, Wiesler D, Fencl M, Saeed T. Fractionation and capillary gas chromatographic-mass spectrometric characterization of the neutral components in marijuana and tobacco smoke condensates. J Chromatogr A. 1982;238(1):141-150. doi:10.1016/S0021-9673(00)82720-X

[4] Wu TC, Tashkin DP, Djahed B, Rose JE; WU TC. Pulmonary hazards of smoking marijuana as compared with tobacco. N Engl J Med. 1988;318(6):347-351. doi:10.1056/NEJM198802113180603 3340105

[5] Tripathi O, Posis AIB, Thompson CA, . In-home cannabis smoking among a cannabis-using convenience sample from the global drug survey: with weighted estimates for US respondents. Cannabis Cannabinoid Res. 2024;9(1):353-362. doi:10.1089/can.2022.0139 36318789

[6] Goodwin RD, Cheslack-Postava K, Santoscoy S, . Trends in cannabis and cigarette use among parents with children at home: 2002 to 2015. Pediatrics. 2018;141(6):e20173506. doi:10.1542/peds.2017-3506 29759986 PMC6317643

[7] Goodwin RD, Kim JH, Cheslack-Postava K, . Trends in cannabis use among adults with children in the home in the United States, 2004-2017: impact of state-level legalization for recreational and medical use. Addiction. 2021;116(10):2770-2778. doi:10.1111/add.15472 33730400

[8] Goodwin RD, Pacek LR, Copeland J, . Trends in daily cannabis use among cigarette smokers: United States, 2002-2014. Am J Public Health. 2018;108(1):137-142. doi:10.2105/AJPH.2017.304050 29161058 PMC5719676

[9] Holitzki H, Dowsett LE, Spackman E, Noseworthy T, Clement F. Health effects of exposure to second- and third-hand marijuana smoke: a systematic review. CMAJ Open. 2017;5(4):E814-E822. doi:10.9778/cmajo.20170112 29192095 PMC5741419

[10] Pope CA III, Burnett RT, Turner MC, . Lung cancer and cardiovascular disease mortality associated with ambient air pollution and cigarette smoke: shape of the exposure-response relationships. Environ Health Perspect. 2011;119(11):1616-1621. doi:10.1289/ehp.1103639 21768054 PMC3226505

[11] Wilson KM, Torok MR, Wei B, . Detecting biomarkers of secondhand marijuana smoke in young children. Pediatr Res. 2017;81(4):589-592. doi:10.1038/pr.2016.261 27911435 PMC5701510

[12] Sangmo L, Braune T, Liu B, . Secondhand marijuana exposure in a convenience sample of young children in New York City. Pediatr Res. 2021;89(4):905-910. doi:10.1038/s41390-020-0958-7 32403116 PMC7882144

[13] Wiegand DM, Methner MM, Grimes GR, . Occupational exposure to secondhand cannabis smoke among law enforcement officers providing security at outdoor concert events. Ann Work Expo Health. 2020;64(7):705-714. doi:10.1093/annweh/wxaa025 32219297 PMC8593821

[14] Wei B, Wang L, Blount BC. Analysis of cannabinoids and their metabolites in human urine. Anal Chem. 2015;87(20):10183-10187. doi:10.1021/acs.analchem.5b02603 26411292 PMC5022557

[15] Wilson KM, Torok MR, Wei B, Wang L, Lowary M, Blount BC. Marijuana and tobacco coexposure in hospitalized children. Pediatrics. 2018;142(6):e20180820. doi:10.1542/peds.2018-0820 30455340 PMC6317534

[16] Posis A, Bellettiere J, Liles S, . Indoor cannabis smoke and children’s health. Prev Med Rep. 2019;14:100853. doi:10.1016/j.pmedr.2019.100853 30976488 PMC6441784

[17] Johnson AB, Wang GS, Wilson K, . Association between secondhand marijuana smoke and respiratory infections in children. Pediatr Res. 2022;91(7):1769-1774. doi:10.1038/s41390-021-01641-0 34321605

[18] Eiden RD, Zhao J, Casey M, Shisler S, Schuetze P, Colder CR. Pre- and postnatal tobacco and cannabis exposure and child behavior problems: bidirectional associations, joint effects, and sex differences. Drug Alcohol Depend. 2018;185:82-92. doi:10.1016/j.drugalcdep.2017.11.038 29428324 PMC5889743

[19] Day NL, Leech SL, Goldschmidt L. The effects of prenatal marijuana exposure on delinquent behaviors are mediated by measures of neurocognitive functioning. Neurotoxicol Teratol. 2011;33(1):129-136. doi:10.1016/j.ntt.2010.07.006 21256427 PMC3052937

[20] Fried PA, Watkinson B. 36- and 48-month neurobehavioral follow-up of children prenatally exposed to marijuana, cigarettes, and alcohol. J Dev Behav Pediatr. 1990;11(2):49-58. doi:10.1097/00004703-199004000-00003 2324288

[21] Sly PD, Flack F. Susceptibility of children to environmental pollutants. Annals of the New York Academy of Sciences. 2008;1140:163-183. doi:10.1196/annals.1454.017 18991915

[22] Au WW. Susceptibility of children to environmental toxic substances. Int J Hyg Environ Health. 2002;205(6):501-503. doi:10.1078/1438-4639-00179 12455272

[23] Landrigan PJ, Goldman LR. Children’s vulnerability to toxic chemicals: a challenge and opportunity to strengthen health and environmental policy. Health Aff (Millwood). 2011;30(5):842-850. doi:10.1377/hlthaff.2011.0151 21543423

[24] Sharma P, Murthy P, Bharath MMS. Chemistry, metabolism, and toxicology of cannabis: clinical implications. Iran J Psychiatry. 2012;7(4):149-156. 23408483 PMC3570572

[25] Hughes SC, Bellettiere J, Nguyen B, . Randomized trial to reduce air particle levels in homes of smokers and children. Am J Prev Med. 2018;54(3):359-367. doi:10.1016/j.amepre.2017.10.017 29305069 PMC5818281

[26] Hovell MF, Bellettiere J, Liles S, ; Fresh Air Research Group. Randomised controlled trial of real-time feedback and brief coaching to reduce indoor smoking. Tob Control. 2020;29(2):183-190. doi:10.1136/tobaccocontrol-2018-054717 30770436 PMC6697236

[27] Klepeis NE, Hughes SC, Edwards RD, . Promoting smoke-free homes: a novel behavioral intervention using real-time audio-visual feedback on airborne particle levels. PLoS One. 2013;8(8):e73251. doi:10.1371/journal.pone.0073251 24009742 PMC3751871

[28] van Buuren S, Groothuis-Oudshoorn K. mice: Multivariate Imputation by Chained Equations in R. J Stat Softw. 2011;45(3):1-67. doi:10.18637/jss.v045.i03

[29] Azur MJ, Stuart EA, Frangakis C, Leaf PJ. Multiple imputation by chained equations: what is it and how does it work? Int J Methods Psychiatr Res. 2011;20(1):40-49. doi:10.1002/mpr.329 21499542 PMC3074241

[30] Willett W, Stampfer MJ. Total energy intake: implications for epidemiologic analyses. Am J Epidemiol. 1986;124(1):17-27. doi:10.1093/oxfordjournals.aje.a114366 3521261

[31] Willett WC, Howe GR, Kushi LH. Adjustment for total energy intake in epidemiologic studies. Am J Clin Nutr. 1997;65(4)(suppl):1220S-1228S. doi:10.1093/ajcn/65.4.1220S 9094926

[32] National Academies of Sciences, Engineering, and Medicine. The Health Effects of Cannabis and Cannabinoids: The Current State of Evidence and Recommendations for Research. National Academies Press; 2017. doi:10.17226/24625 28182367

[33] Wei B, Smith DM, Travers MJ, O’Connor RJ, Goniewicz ML, Hyland AJ. Chapter One - Secondhand marijuana smoke (SHMS): Exposure occurrence, biological analysis and potential health effects. In: Fishbein JC, Heilman JM, eds. Advances in Molecular Toxicology. Vol 13. Elsevier; 2019:1-30. doi:10.1016/B978-0-444-64293-6.00001-4

[34] Tripathi O, Bellettiere J, Liles S, Shi Y. Location and home rules of cannabis use—findings from marijuana use and environmental survey 2020, a nationally representative survey in the United States. Prev Med Rep. 2023;35:102289. doi:10.1016/j.pmedr.2023.102289 37408996 PMC10319339

[35] Hennessy M, Bleakley A, Mallya G, Romer D. The effect of household smoking bans on household smoking. Am J Public Health. 2014;104(4):721-727. doi:10.2105/AJPH.2013.301634 24524533 PMC4025695

[36] US Department of Health and Human Services. Tobacco Use: Smoke-Free Policies. Healthy People 2030 resource page. November 2012. Accessed February 11, 2024. https://health.gov/healthypeople/tools-action/browse-evidence-based-resources/tobacco-use-smoke-free-policies

[37] Caron KT, Zhu W, Bernert JT, . Geometric mean serum cotinine concentrations confirm a continued decline in secondhand smoke exposure among US nonsmokers—NHANES 2003 to 2018. Int J Environ Res Public Health. 2022;19(10):5862. doi:10.3390/ijerph19105862 35627398 PMC9140715

[38] Goodwin RS, Darwin WD, Chiang CN, Shih M, Li SH, Huestis MA. Urinary elimination of 11-nor-9-carboxy-delta^9^-tetrahydrocannnabinol in cannabis users during continuously monitored abstinence. J Anal Toxicol. 2008;32(8):562-569. doi:10.1093/jat/32.8.562 19007504 PMC2587336

[39] Ott WR, Zhao T, Cheng KC, Wallace LA, Hildemann LM. Measuring indoor fine particle concentrations, emission rates, and decay rates from cannabis use in a residence. Atmos Environ X. 2021;10:100106. doi:10.1016/j.aeaoa.2021.100106

[40] Strong DR, Myers MG, Pulvers K, Noble M, Brikmanis K, Doran N. Marijuana use among US tobacco users: findings from wave 1 of the population assessment of tobacco health (PATH) study. Drug Alcohol Depend. 2018;186:16-22. doi:10.1016/j.drugalcdep.2017.12.044 29529455

[41] Klepeis NE, Bellettiere J, Hughes SC, . Fine particles in homes of predominantly low-income families with children and smokers: key physical and behavioral determinants to inform indoor-air-quality interventions. PLoS One. 2017;12(5):e0177718. doi:10.1371/journal.pone.0177718 28545099 PMC5435241

