# Supplementary material for: Exposure to Secondhand Cannabis Smoke Among Children
Source: JAMA Netw Open. 2025 Jan 23;8(1):e2455963. doi: 10.1001/jamanetworkopen.2024.55963 (PMC11758598; doi:10.1001/jamanetworkopen.2024.55963)

Supplemental Online Content

Tripathi O, Parada H, Sosnoff C, et al. Exposure to secondhand cannabis smoke among children. *JAMA Netw Open.* 2025;8(1):e2455963.
doi:10.1001/jamanetworkopen.2024.55963

eMethods. Questionnaire, Nicotine Dosimeter Data, and Sensitivity Analyses

eTable 1. Models for Addressing Variance in Air Particle Data Due to Various Particle Generating Activities

eTable 2. Descriptive Statistics for Reported Air Particle Generating and Ventilating Events and Air Nicotine

eTable 3. Linear Regression for Total THC Equivalents Among Those With Detectable Urinary Cannabinoids

eTable 4. Age-Stratified Logistic Regression of Total Detectable THC Equivalents in Urine of Children

eTable 5. Descriptive Statistics for Demographic Variables Stratified by Missingness on Reported In-Home Cannabis Smoking

eTable 6. Logistic Regression of Total Detectable THC Equivalents in Children, Among Those With Data on Reported In-Home Cannabis Smoking

eFigure 1. Restricted Cubic Spline Regression between Number of Daily Nonspecific Smoking Events and Total THC Equivalents

eFigure 2. Distribution of Log-TTE Among Households Reporting In-Home Cannabis Smoking Among Children With Detectable Levels of TTE

eFigure 3. Distribution of Log-TTE by Number of Daily Nonspecific Smoking Events, Among Children With Detectable Levels of TTE

eFigure 4. Distribution of Log-TTE by Ascertained Number of Daily Cannabis Smoking Events, Among Children With Detectable Levels of TTE

This supplemental material has been provided by the authors to give readers additional information about their work.

© 2025 Tripathi O et al. *JAMA Network Open*.

eMethods. Questionnaire, Nicotine Dosimeter Data, and Sensitivity Analyses

Questionnaire data

1. **Demographics:** The questionnaire included questions on the sex of the child (male or female), age of the child (years), parent/guardian's education (years), family income (increments of $10,000), race/ethnicity (Black, Hispanic, White, Other (Asian, Native American, Pacific Islander, mixed, unspecified), and type of home (apartment/condominium, detached house, or other).
2. **Report of in-home tobacco smoking:** Parents/guardians were asked to report whether cigarettes, cigars, pipe tobacco, hookah or e-cigarettes were smoked inside the home in the past 7 days (Yes/No, for each product).
3. **Report of particle-generating activities:** For each in-home *particle-generating* activity in the past 7 days, parents/guardians were asked the number of times incense was burned; food was burned; participants fried food with oil; gas or propane appliances were used to cook or heat the home; aerosol spray products were used; participants vacuumed, dusted, or swept; and anything else that generated air particles. Participants were also asked about burning wood and using any gas/space/wall-mounted heating device in the past 7 days. For each activity 3 factors were measured: (1) number of days each activity was conducted in-home, (2) average number of hours per day each activity was conducted, and (3) intensity of activity [1(low), 2(medium), 3(high)]. The responses from each factor were multiplied to create an overall assessment of the total impact of wood burning or use of heater in the past 7 days (Particle-Generating Activity Impact Score). Each of these composite variables is unitless.
4. **Report of particle ventilating activities:** Parents/guardians were asked about the frequency of engaging in in-home ventilation activities during in-home particle generating activities in the past 7 days: Questions were asked about opening a window, opening an interior door, opening an exterior door, use of air purifier, use of exhaust fan, use of ceiling fan, use of window fan or AC, and use of central HVAC system, during particle generating activities (cooking, cleaning or smoking). The responses for these activities (cooking, cleaning, smoking) were coded as: during all three of the activities [3], during two of three activities [2], during only one activity [1], and during none of the activities [0]. Additionally, participants were asked about central HVAC use, air purifier use, exhaust fan use, and window fan or AC use in the past 7 days. For each activity 3 factors were measured: (1) number of days each activity was conducted in home, (2) average number of hours per day each activity was conducted, and (3) intensity of activity [1(low), 2(medium), 3(high)]. The responses from each factor, for each activity, were multiplied to create an overall assessment of the total impact of these ventilating activities in the past 7 days (Particle Ventilation Activity Impact Score). Each of these composite variables is unitless.

Nicotine Dosimeter

Average air nicotine concentration ($\mu g/m^3$) was estimated via nicotine dosimeter assays conducted using liquid chromatography/tandem mass spectrometry with electro-spray ionization. This captures information about in-home tobacco smoking and e-cigarette use that may not have been caught through the questionnaire.

Descriptive statistics for demographics are presented in Table 1. Descriptive statistics on reported tobacco smoking, particle-generating activities, particle ventilating activities and nicotine dosimeter are presented in eTable 3.

Sensitivity Analyses:

To assess the effect of our statistical treatment of values below LOD, log-linear regression models were repeated after replacing LOD/2 (0.0025 ng/ml) with (a) LOD/sqrt(2) (0.0035 ng/ml), and (b) zero. Results were not sensitive to how values below the LOD were treated. (eTable 3).

Among those with detectable levels of TTE, to provide clarity when interpreting results, we provide a box-plot showing the distribution of TTE between households with and without report of in-home cannabis smoking, and two scatter plots with fitted regression line (95%CI) for TTE against 1) number of daily non-specified smoking events and 2) Ascertained number of daily cannabis smoking events (eFigure 2, 3, 4, respectively).

eTable 1. Models for Addressing Variance in Air Particle Data Due to Various Particle Generating Activities

Model	Relation expressed	Output	Action
Model A	*Number of daily smoking events (>= 15,000 counts/0.01ft^3 over 5 minutes)* = air nicotine + reported tobacco smoking + reported other indoor particle generating activities + reported ventilation activities during indoor particle generating activities	Residual (Model A) for each participant	Residual (Model A) - Residual (Model B) = Ascertained number of daily cannabis smoking events
Model B	*Number of daily smoking events (>= 15,000 counts/0.01ft^3 over 5 minutes)* = air nicotine + reported indoor tobacco smoking + reported other indoor particle generating activities + reported ventilation activities during indoor particle generating activities + **reported in-home cannabis smoking**	Residual (Model B) for each participant	

© 2025 Tripathi O et al. *JAMA Network Open*.

eTable 2. Descriptive Statistics for Reported Air Particle Generating and Ventilating Events and Air Nicotine

	Baseline (n=275)	
Characteristics	n	%
Air nicotine (ug/m^3)		
mean (sd)	0.4 (1.6)	
median (range)	0.0 (0.0, 15.7)	
Missing	12	4.4%
HVAC use Impact Score		
mean (sd)	22.8 (67.0)	
median (range)	0.0 (0.0, 504.0)	
Air Purifier Use Impact Score		
mean (sd)	14.0 (64.0)	
median (range)	0.0 (0.0, 504.0)	
Exhaust Fan Use Impact Score		
mean (sd)	13.7 (50.1)	
median (range)	2.0 (0.0, 504.0)	
AC Use Impact Score		
mean (sd)	62.6 (141.9)	
median (range)	0.0 (0.0, 504.0)	
Burning Wood Impact Score		
mean (sd)	2.3 (12.0)	
median (range)	0.0 (0.0, 105.0)	
Gas Heater Use Impact Score		
mean (sd)	4.7 (23.0)	
median (range)	0.0 (0.0, 252.0)	
Number of times incense burned used in past 7 days		
mean (sd)	5.4 (17.0)	
median (range)	0.0 (0.0, 168.0)	
Number of times food burned used in past 7 days		
mean (sd)	1.6 (4.0)	
median (range)	0.0 (0.0, 30.0)	
Number of times oil fried in past 7 days		
mean (sd)	17.0 (24.4)	
median (range)	9.0 (0.0, 196.0)	
Number of times gas/propane appliance used in past 7 days		
mean (sd)	22.6 (30.2)	
median (range)	14.0 (0.0, 147.0)	
Number of times electric appliance used to cook/heat in past 7 days		
mean (sd)	44.6 (59.3)	
median (range)	28.0 (0.0, 700.0)	
Number of times aerosol spray products used in past 7 days		

© 2025 Tripathi O et al. *JAMA Network Open*.

mean (sd)		20.3 (58.0)
median (range)		4.0 (0.0, 672.0)
Number of times vacuumed/dusted/swept in past 7 days		
mean (sd)		17.8 (23.5)
median (range)		9.0 (0.0, 147.0)
Number of times do anything else that generates particles in past 7 days		
mean (sd)		4.9 (20.7)
median (range)		0.0 (0.0, 196.0)
Number of times do anything else that generates particles in past 7 days		
mean (sd)		2.3 (19.3)
median (range)		0.0 (0.0, 245.0)
Cigarettes smoked inside home in past 7 days		
No	192	69.8%
Yes	49	17.8%
Missing	34	12.4%
Cigar smoked inside home in past 7 days		
No	230	83.5%
Yes	11	4.0%
Missing	34	12.4%
Pipe tobacco smoked inside home in past 7 days		
No	238	86.5%
Yes	3	1.1%
Missing	34	12.4%
Hookah smoked inside home in past 7 days		
No	238	86.5%
Yes	3	1.1%
Missing	34	12.4%
e-cigarette smoked inside home in past 7 days		
No	203	73.8%
Yes	38	13.8%
Missing	34	12.4%
Did anyone open windows to room with cooking, cleaning, or smoking		
No	36	13.1%
1	36	13.1%
2	133	48.4%
3 (during all three activities)	70	25.4%
Missing	0	0.0%
Did anyone close interior doors to room with cooking, cleaning, or smoking		
No	133	48.3%
1	72	26.2%
2	53	19.3%
3 (all three activities)	17	6.2%
Missing	0	0.00%
Did anyone open exterior doors to room with cooking, cleaning, or smoking		

© 2025 Tripathi O et al. *JAMA Network Open*.

No	36	13.1%
1	35	12.7%
2	111	40.4%
3 (all three activities)	93	33.8%
Missing	0	0.0%
Did anyone use air purifier with a fan in the room with cooking, cleaning, or smoking		
No	257	93.5%
Yes	13	4.7%
Missing	5	1.8%
Did anyone use exhaust fan in the room with cooking, cleaning, or smoking		
No	113	41.0%
1	103	37.5%
2	44	16.0%
3 (all three activities)	15	5.5%
Missing	0	0.0%
Did anyone use a ceiling fan in the room with cooking, cleaning, or smoking		
No	103	37.5%
1	49	17.8%
2	93	33.8%
3 (all three activities)	30	10.9%
Missing	0	0.0%
Did anyone use a window fan or AC in the room with cooking, cleaning, or smoking		
No	218	79.3%
Yes	54	19.6%
Missing	3	1.1%
Did anyone use a central HVAC system in the room with cooking, cleaning, or smoking		
No	221	80.4%
Yes	49	17.8%
Missing	5	1.8%

© 2025 Tripathi O et al. *JAMA Network Open*.

eTable 3. Linear Regression for Total THC Equivalents Among Those With Detectable[a] Urinary Cannabinoids

	LOD/sqrt(2)		<LOD as zero	
	linear (log outcome)		linear (log outcome)	
	%	95% CI	%	95% CI
**In-home cannabis smoking in past 7 days[b]**				
M1	77.5	-0.3, 215.8	91.2	-1.6, 271.4
M2	73.2	-3.9, 212.4	86.1	-5.2, 265.2
M3	82.4	-1.3, 237.3	97.3	-2.5, 299.1
**Number of daily smoking events[c]**				
M1	6.3	2.1, 10.8	7.3	2.3, 12.4
M2	4.8	-0.1, 9.8	5.3	-0.3, 11.1
M3	4.6	-0.4, 9.8	5.2	-0.6, 11.2
**Ascertained number of daily cannabis smoking events[d]**				
M1	19.7	-15.3, 69.1	22.4	-17.8, 82.2
M2	30.7	-8.5, 86.6	36.2	-9.3, 104.7
M3	34.2	-7.0, 93.6	40.1	-7.9, 113.3

[a] detectability of TTE was determined based on cannabis biomarkers <LOD treated as LOD/sqrt(2), and as zero
[b] Reported by the parent/guardian
[c] Determined by a validated air particle count algorithm, using air particle data non-specific to the source of air particles (e.g., smoking cigarettes, smoking cannabis, burning toast, cooking with oil, burning incense)
[d] Ascertained by residualization, adjusting for air nicotine, tobacco smoking, and other reported air particle generating/ventilating activities
M1: unadjusted model
M2: M1 model + the sex of the child (male or female), age of the child (years), parent/guardian's education (years), family income (increments of $10,000), race/ethnicity (Black, Hispanic, White, Other (Asian, Native American, Pacific Islander, mixed, unspecified)
M3: M2 model + type of home (apartment/condominium, detached house, or other)

© 2025 Tripathi O et al. *JAMA Network Open*.

eTable 4. Age-Stratified Logistic Regression of Total Detectable THC Equivalents in Urine of Children

	Children < 6 years old		Children 6+ years old	
	Binomial (n=208)		Binomial (n=67)	
	OR	95% CI	OR	95% CI
In-home cannabis smoking in past 7 days (yes/no)[a]				
M1	4.63	2.12, 10.12	8.82	1.99, 39.27
M2	3.61	1.55, 8.41	72.71	4.10, 1288.23
M3	3.65	1.56, 8.52	115.07	5.05, 2621.23
Number of daily (non-specific) smoking events[b]				
M1	1.11	1.04, 1.18	1.18	1.03, 1.35
M2	1.08	1.00,1.16	1.21	1.03, 1.42
M3	1.08	1.01, 1.16	1.23	1.04, 1.45
Ascertained number of daily cannabis smoking events[c]				
M1	2.23	1.37, 3.65	2.68	1.11, 6.45
M2	2.19	1.30, 3.66	8.37	1.89, 37.08
M3	2.17	1.29, 3.65	8.13	1.87, 35.30

a Reported by the parent/guardian; the “Other” race/ethnicity category comprises: Asian, Native American, Pacific Islander, mixed, unspecified
b Determined by a validated air particle count algorithm, using air particle data non-specific to the source of air particles (e.g., smoking cigarettes, smoking cannabis, burning toast, cooking with oil, burning incense)
c Ascertained by residualization, adjusting for air nicotine, tobacco smoking, and other air reported particle generating/ventilating activities
M1: unadjusted model
M2: M1 model + the sex of the child (male or female), age of the child (years), parent/guardian’s education (years), family income (increments of $10,000), race/ethnicity (Black, Hispanic, White, Other (Asian, Native American, Pacific Islander, mixed, unspecified)
M3: M2 model + type of home (apartment/condominium, detached house, or other)
* For both procedures, detectability of TTE was determined based on cannabis biomarkers <LOD treated as half of LOD (0.0025 ng/ml)

© 2025 Tripathi O et al. *JAMA Network Open*.

eTable 5. Descriptive Statistics for Demographic Variables Stratified by Missingness on Reported In-Home Cannabis Smoking

	Not missing data on "Cannabis smoked inside home in the last 7 days" (n=198)		Missing data on "Cannabis smoked inside home in the last 7 days" (n=77)	
Characteristics	n	%	n	%
Cannabis smoked inside home in last 7 days				
No	169	85.35%	0	0.00%
Yes	29	14.65%	0	0.00%
Missing	0	0.00%	0	0.00%
Number of daily smoking events				
mean (sd)	2.63 (4.62)		3.57 (5.82)	
median (range)	0.86 (0.00, 33.56)		0.83 (0.00, 24.20)	
Total THC Equivalents[a,b] (nmol/L)				
Not detected	138	69.70%	62	80.52%
Detected	60	30.30%	15	19.50%
mean (sd) [half LOD][c]	0.14 (0.4)		0.10 (0.22)	
median (range) [half LOD]	0.02 (0.02, 4.95)		0.02 (0.02, 1.21)	
THC				
Not detected	179	90.40%	72	93.51%
Detected	19	9.60%	5	6.49%
OH-THC				
Not detected	155	78.28%	63	81.82%
Detected	41	20.71%	13	16.88%
Missing	2	1.01%	1	1.30%
COOOH-THC				
Not detected	146	73.74%	63	81.82%
Detected	49	24.75%	13	16.88%
Missing	3	1.52%	1	1.30%
Age of child				
mean (sd)	3.72 (3.72)		3.25 (3.13)	
median (range)	3.00 (0.00, 14.00)		3.00 (0.00, 12.00)	
Sex of child				
Female	106	53.54%	39	50.65%
Male	92	46.46%	38	49.35%
Race/ethnicity of child				
Black	28	14.14%	10	12.99%
White	102	51.52%	30	38.96%
Hispanic	34	17.17%	19	24.68%
Other	34	17.17%	18	23.38%

© 2025 Tripathi O et al. *JAMA Network Open*.

Family Income (annual)				
less than $10,000	41	20.71%	12	15.58%
$10,000 - $19,999	33	16.67%	11	14.29%
$20,000 - $29,999	36	18.18%	10	12.99%
$30,000 - $39,999	26	13.13%	6	7.79%
$40,000 - $49,999	17	8.59%	9	11.69%
$50,000 - $59,999	7	3.54%	5	6.49%
$60,000 - $69,999	5	2.53%	4	5.19%
$70,000 - $79,999	2	1.01%	8	10.39%
$80,000 - $89,999	3	1.52%	1	1.30%
$90,000 - $99,999	2	1.01%	4	5.19%
$100,000 or more	4	2.02%	7	9.09%
Missing	22	11.11%		
Years of education of parent				
mean (sd)	13.16 (2.79)		13.11 (4.41)	
median (range)	13.00 (2.00, 22.00)		13.00 (0.70, 22.00)	
Missing	2			
Home Type				
Apartment/Condo	85	42.93%	29	37.66%
Detached House	91	45.96%	28	36.36%
Other	22	11.11%	20	25.97%

[a] Each biomarker (ng/ml) was divided by its molecular weight (ng/nmol) to equal nmol/ml and summed. TTE = [THC (ng/ml)/314.5 (ng/nmol)] + [OH-THC(ng/ml)/330.5 (ng/nmol)] + [COOH-THC (ng/ml)/344.4 (ng/nmol)]
[b] Those with undetectable LOD for all three biomarkers were considered 'not detected' for TTE
[c] half LOD = 0.0025 ng/ml

© 2025 Tripathi O et al. *JAMA Network Open*.

eTable 6. Logistic Regression of Total Detectable THC Equivalents in Children, Among Those With Data on Reported In-Home Cannabis Smoking

	Binomial (n=198)	
	OR	95% CI
In-home cannabis smoking in past 7 days[a]		
M1	7.17	2.10, 17.74
M2	5.97	2.25, 17.10
M3	6.24	2.33, 18.03
Number of daily (non-specific) smoking events[b]		
M1	1.17	1.08, 1.27
M2	1.12	1.03, 1.23
M3	1.12	1.03,1.24
Ascertained number of daily cannabis smoking events[c]		
M1	3.55	1.70, 7.73
M2	3.32	1.45, 7.87
M3	3.35	1.46, 7.95

[a] Reported by the parent/guardian; the "Other" race/ethnicity category comprises: Asian, Native American, Pacific Islander, mixed, unspecified

[b] Determined by a validated air particle count algorithm, using air particle data non-specific to the source of air particles (e.g., smoking cigarettes, smoking cannabis, burning toast, cooking with oil, burning incense)

[c] Ascertained by residualization, adjusting for air nicotine, tobacco smoking, and other air reported particle generating/ventilating activities

M1: unadjusted model

M2: M1 model + the sex of the child (male or female), age of the child (years), parent/guardian's education (years), family income (increments of $10,000), race/ethnicity (Black, White, Hispanic, or Other)

M3: M2 model + type of home (apartment/condominium, detached house, or other)

* For both procedures, detectability of TTE was determined based on cannabis biomarkers <LOD treated as half of LOD (0.0025 ng/ml)

eFigure 1. Restricted Cubic Spline Regression Between Number of Daily Nonspecific Smoking Events and Total THC Equivalents.

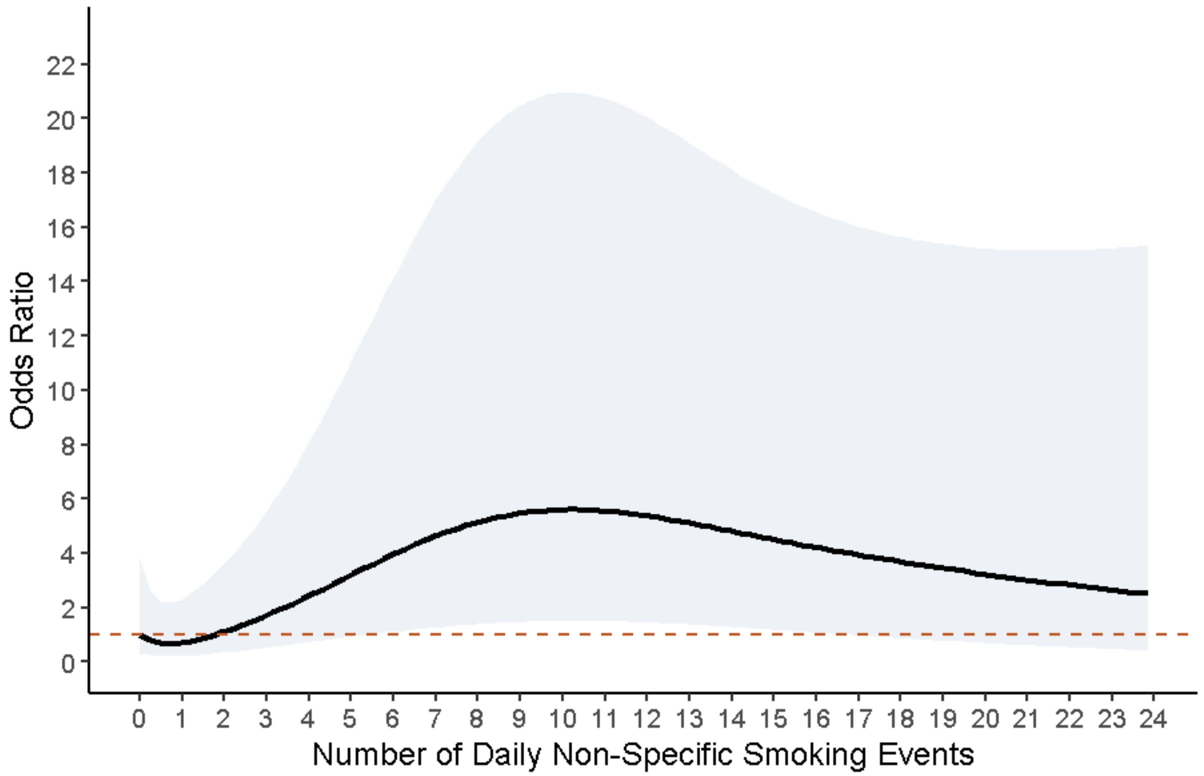

eFigure 2. Distribution of Log-TTE Among Households Reporting In-Home Cannabis Smoking Among Children With Detectable Levels of TTE

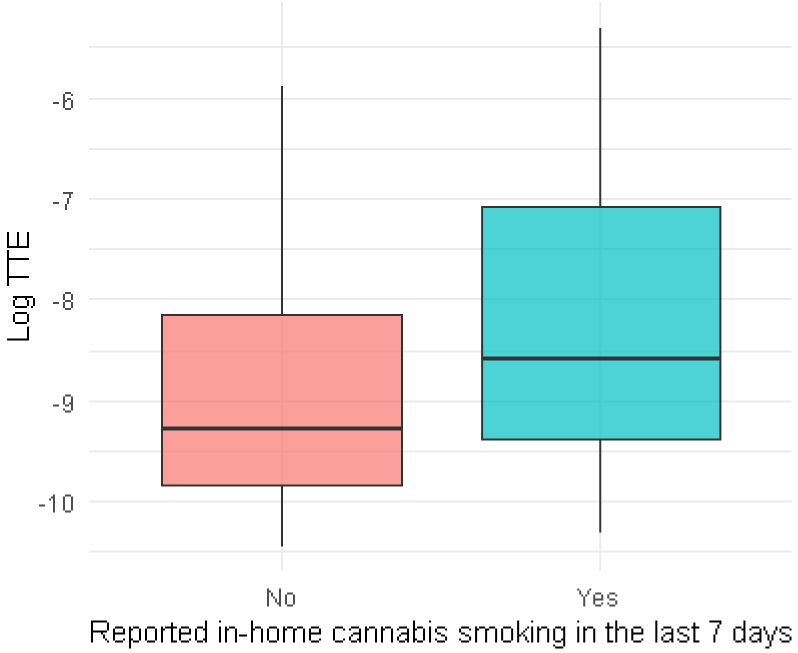

eFigure 3. Distribution of Log-TTE by Number of Daily Nonspecific Smoking Events, Among Children With Detectable Levels of TTE

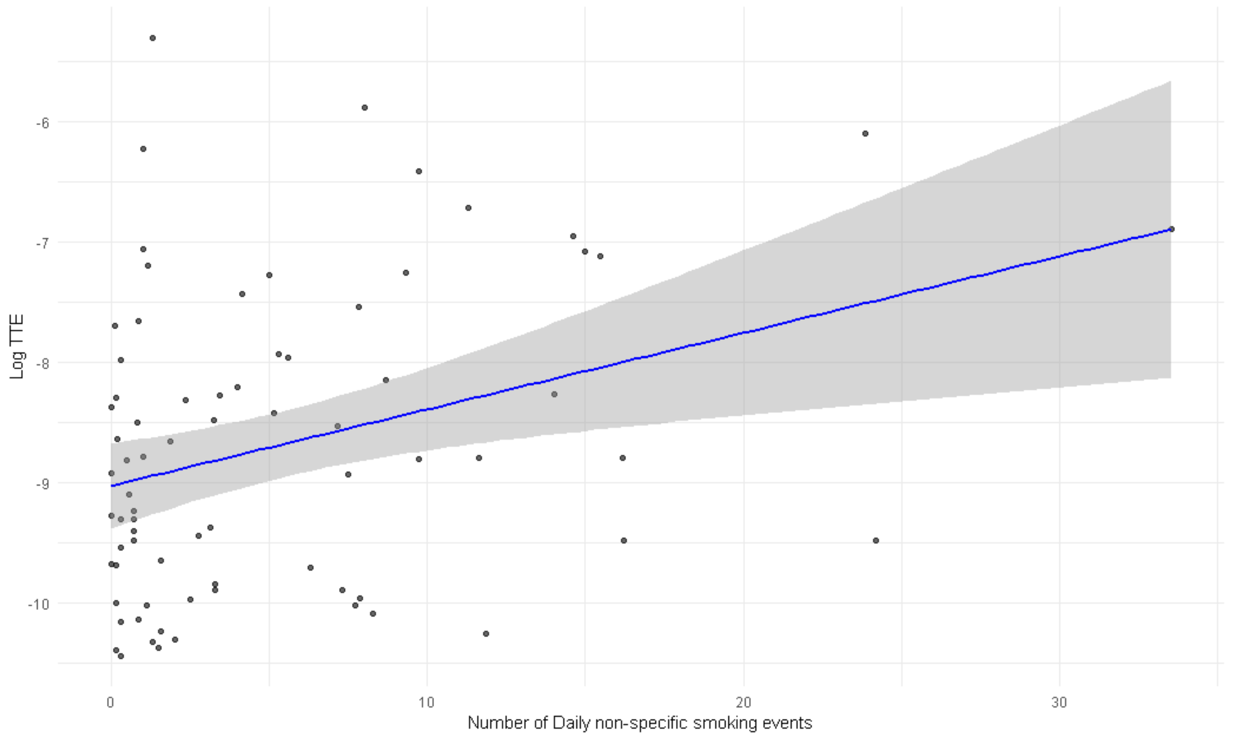

eFigure 4. Distribution of Log-TTE by Ascertained Number of Daily Cannabis Smoking Events, Among Children With Detectable Levels of TTE

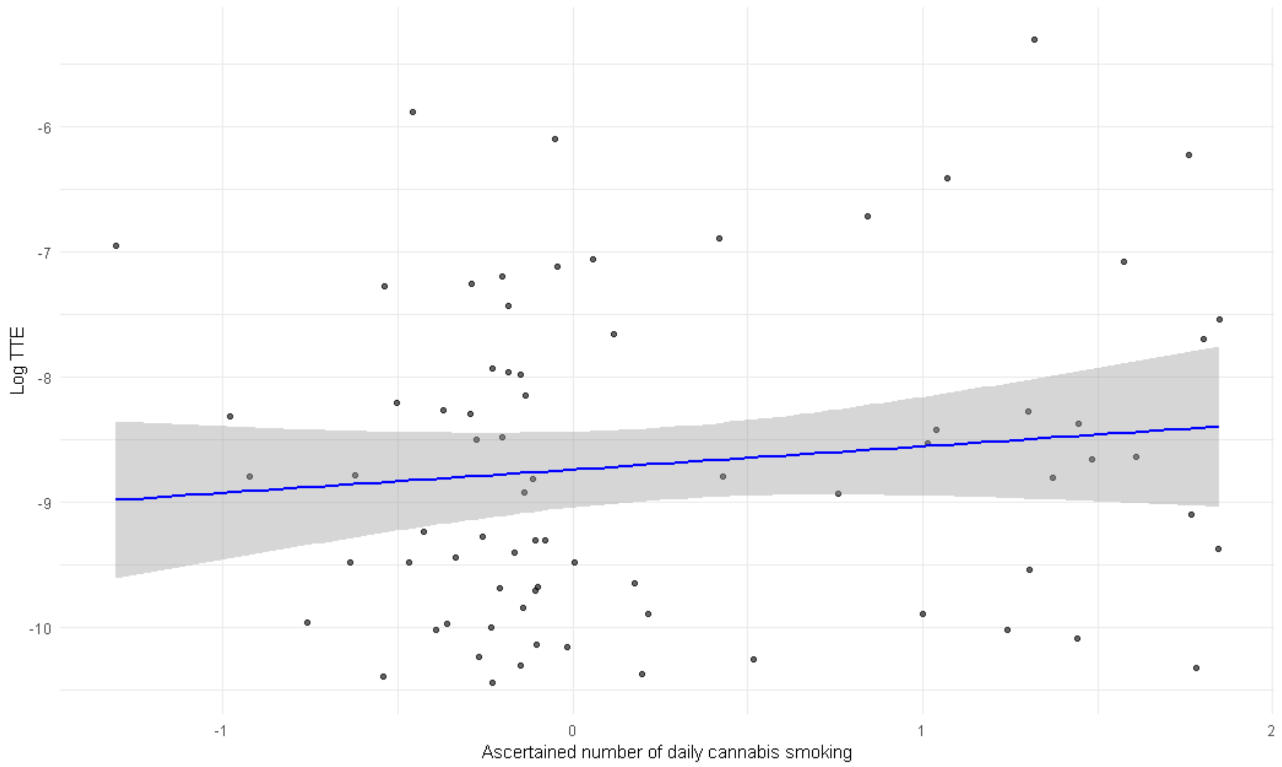

Supplement: Supplement 1. — eMethods. Questionnaire, Nicotine Dosimeter Data, and Sensitivity Analyses eTable 1. Models for Addressing Variance in Air Particle Data Due to Various Particle Generating Activities eTable 2. Descriptive Statistics for Reported Air Particle Generating and Ventilating Events and Air Nicotine eTable 3. Linear Regression for Total THC Equivalents Among Those With Detectable Urinary Cannabinoids eTable 4. Age-Stratified Logistic Regression of Total Detectable THC Equivalents in Urine of Children eTable 5. Descriptive Statistics for Demographic Variables Stratified by Missingness on Reported In-Home Cannabis Smoking eTable 6. Logistic Regression of Total Detectable THC Equivalents in Children, Among Those With Data on Reported In-Home Cannabis Smoking eFigure 1. Restricted Cubic Spline Regression between Number of Daily Nonspecific Smoking Events and Total THC Equivalents eFigure 2. Distribution of Log-TTE Among Households Reporting In-Home Cannabis Smoking Among Children With Detectable Levels of TTE eFigure 3. Distribution of Log-TTE by Number of Daily Nonspecific Smoking Events, Among Children With Detectable Levels of TTE eFigure 4. Distribution of Log-TTE by Ascertained Number of Daily Cannabis Smoking Events, Among Children With Detectable Levels of TTE [file jamanetwopen-e2455963-s001.pdf]
