# Supplementary material for: Exposure to Secondhand Cannabis Smoke Among Children
Source: JAMA Netw Open. 2025 Jan 23;8(1):e2455963. doi: 10.1001/jamanetworkopen.2024.55963 (PMC11758598; doi:10.1001/jamanetworkopen.2024.55963)
Supplement: Supplement 2. — Data Sharing Statement [file jamanetwopen-e2455963-s002.pdf]

Data Sharing Statement

Tripathi. Exposure to Secondhand Cannabis Smoke Among Children. *JAMA Netw Open*. Published January 23, 2025. doi:10.1001/jamanetworkopen.2024.55963

Data
Data available: Yes
Data types: Deidentified participant data, Data dictionary
How to access data: Our reserved Digital Object Identifier (not yet activated) for the archive is: https://doi.org/10.6075/J0R49R4Z The tentative citation for the archive is: Bellettiere, John; Liles, Sandy; Hovell, Melbourne F (2024). In-Home Tobacco and Cannabis Smoking and Secondhand Exposure of Children: Evidence from Urinary Biomarkers. UC San Diego Library Digital Collections. Our archived statistical dataset will be available at a Restricted Access level of security. The initial request for access must be made to the senior author, John Bellettiere, who is also a corresponding author on this manuscript. His email address is jbellettiere1@gmail.com.
When available: With publication

Supporting Documents
Document types: Informed consent form, Other (please specify)
Additional Information: Published journal articles based on data from the two research projects; interview codebook; study protocols; approvals of study procedures; reports made to official supervisory bodies
How to access documents: These documents are all contained in a well-organized data archive that the authors were able to provide thanks to the collaboration of University of California San Diego Library archive curators. The Digital Object Identifier for the archive (not to be activated until publication of this manuscript) is: https://doi.org/10.6075/J0R49R4Z All data associated with the archive except the statistical dataset will be released when the manuscript is published, and the link to the archive will be included in the manuscript. Those wishing to also access the statistical dataset can email Dr. John Bellettiere at his email address (jbellettiere1@gmail.com), which is provided within the archive, to request access.
When available: With publication

Additional Information
Who can access the data: researchers whose proposed use of the statistical dataset has been approved
Types of analyses: For investigation of an academic research question
Mechanisms of data availability: If the applicant researcher's proposed use of the statistical dataset is approved by the principal investigator, John Bellettiere, he will provide the applicant with a link to access the dataset
Any additional restrictions: None
